# Supplementary material for: Functional organization of the neonatal thalamus across development depicted by functional MRI
Source: Imaging Neurosci (Camb). 2025 Jul 24;3:IMAG.a.87. doi: 10.1162/IMAG.a.87 (PMC12330859; doi:10.1162/IMAG.a.87)
Supplement: Supplementary Material [file IMAG.a.87_supp.pdf]

1 Supplementary Materials

1.1 dHCP protocol exclusion criteria

The dHCP exclusion criteria include infants or mothers with contraindications to MRI, preterm infants medically unfit for scanning, or cases where language barriers impeded consent. In addition, dHCP provides QC annotations for functional MRI data. Specifically, we excluded all subjects for whom the qc_fmri_flagged variable was set to True, indicating issues detected during preprocessing. Common failure reasons included excessive motion (DVARS), poor distortion correction (FMAP), low temporal signal-to-noise ratio (tSNR), registration failures (standard-to-structural transformation), corrupted spin-echo images, or a combination of these elements.

1.2 Choice of the number of clusters

The choice of k=5 in K-means is based on several points:

(1) We wanted to have a similar number of clusters for all age groups to be able to follow the different clusters and their respective connectivity pattern changes, and hence we could not choose "the optimal" cluster for each age group.

(2) We suppose that stability is an indirect measure to the optimality of a cluster number. In other words, if the variability of the clusters generated using different (timepoints or subject) bootstraps is low for a certain cluster number, then it would mean that this cluster number is a good fit. However, this variability should in general (i) increase by increasing the cluster number (i.e. one can think about the upper bound case of assigning a cluster to each voxel), and (ii) decrease when the number of subjects increases for a certain age-bin.

From this perspective, we would like a number of clusters that is high enough, but also not variable and more stable. Looking at Supplementary Figure S2, we observe that the gap in variability increases for a number of clusters above 4. Especially from 5 to 6 clusters, if we look at the most represented age-bins (41 and 42 weeks with N=113 and N=106 respectively). The choice for each age group is shown in Supplementary Figure 14 (column Cov_IoU - subjects), where a similar reasoning was also applied to the timepoints bootstraps (column Cov_IoU - time) by analyzing the patterns of Supplementary Figure S1

(3) The silhouette score which quantifies the proximity between data points and there assigned cluster compared to other clusters (the higher the better) showed different trends that are highlighted in

Supplementary Figure S4 (column Silhouette Score) where a general low standard deviation (in subjects bootstraps) for 5 clusters was observed.

Based on these 3 elements: a fixed number of clusters across all age groups, subjects and timepoints bootstraps trends and the silhouette score (Supplementary Figure S3), the cluster number that had the highest number of votes across the different age groups was k=5.

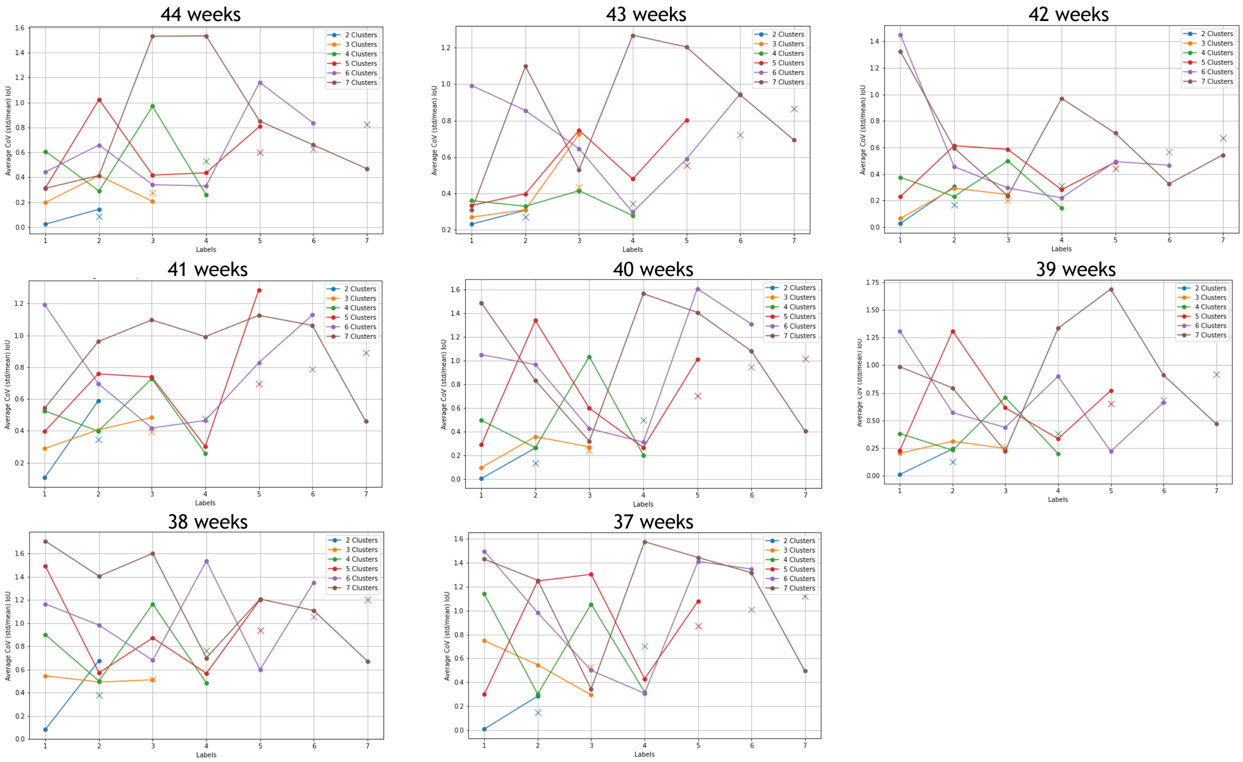

Figure S1: Variability through *timepoints bootstraps* for all age groups (N=25).

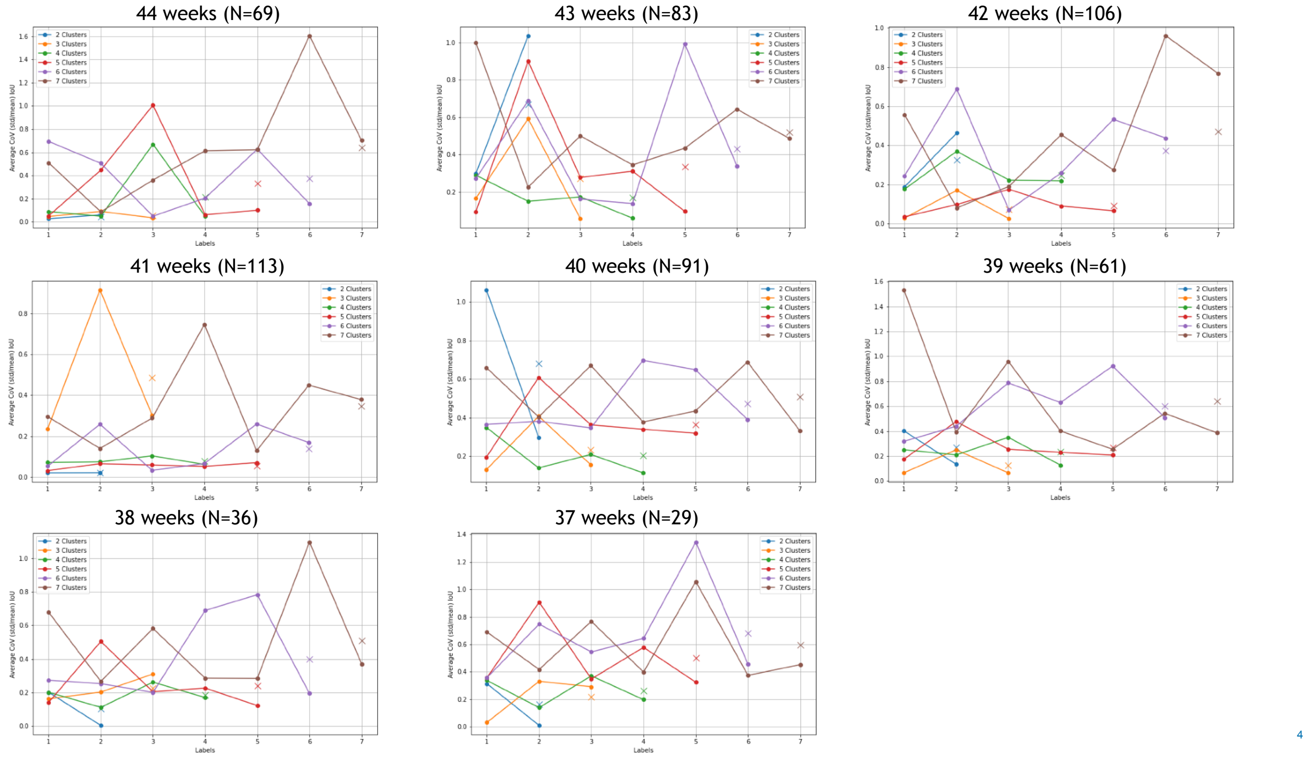

Figure S2: Variability through *subjects bootstraps* for all age groups. The number of subjects per age group is shown on the top.

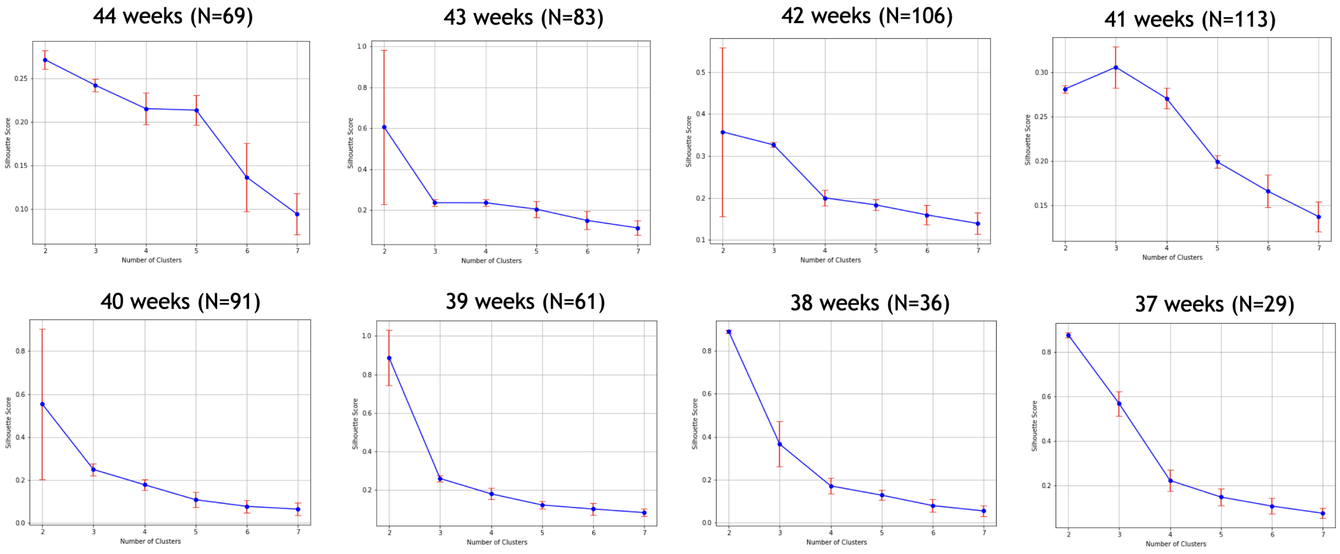

Figure S3: Silhouette score for the different cluster numbers (k) across all age groups. The number of subjects per age group is shown on the top.

Table S1: Mapping of the 40 seed ROIs and their respective abbreviations used throughout the manuscript. The last four rows correspond to the thalamic labels that were merged to define thalamic voxels.

ROI Label Name in dHCP	ROI Abbreviation
Hippocampus left	Hippocampus (L)
Hippocampus right	Hippocampus (R)
Amygdala left	Amygdala (L)
Amygdala right	Amygdala (R)
Anterior temporal lobe, medial part left GM	Anterior temporal cortex (ML)
Anterior temporal lobe, medial part right GM	Anterior temporal cortex (MR)
Anterior temporal lobe, lateral part left GM	Anterior temporal cortex (LL)
Anterior temporal lobe, lateral part right GM	Anterior temporal cortex (LR)
Gyri parahippocampalis et ambiens anterior part left GM	Parahippocampal gyrus (AL)
Gyri parahippocampalis et ambiens anterior part right GM	Parahippocampal gyrus (AR)
Superior temporal gyrus, middle part left GM	Superior temporal gyrus (MiL)
Superior temporal gyrus, middle part right GM	Superior temporal gyrus (MiR)
Medial and inferior temporal gyri anterior part left GM	Medial & Inferior temporal gyri (AL)
Medial and inferior temporal gyri anterior part right GM	Medial & Inferior temporal gyri (AR)
Lateral occipitotemporal gyrus, gyrus fusiformis anterior part left GM	Gyrus fusiformis (AL)
Lateral occipitotemporal gyrus, gyrus fusiformis anterior part right GM	Gyrus fusiformis (AR)
Cerebellum left	Cerebellum (L)
Cerebellum right	Cerebellum (R)
Insula right GM	Insula (R)
Insula left GM	Insula (L)
Occipital lobe right GM	Occipital cortex (R)
Occipital lobe left GM	Occipital cortex (L)
Gyri parahippocampalis et ambiens posterior part right GM	Parahippocampal gyrus (PR)
Gyri parahippocampalis et ambiens posterior part left GM	Parahippocampal gyrus (PL)
Lateral occipitotemporal gyrus, gyrus fusiformis posterior part right GM	Gyrus fusiformis (PR)
Lateral occipitotemporal gyrus, gyrus fusiformis posterior part left GM	Gyrus fusiformis (PL)
Medial and inferior temporal gyri posterior part right GM	Medial & Inferior temporal gyri (PR)
Medial and inferior temporal gyri posterior part left GM	Medial & Inferior temporal gyri (PL)
Superior temporal gyrus, posterior part right GM	Superior temporal gyrus (PR)
Superior temporal gyrus, posterior part left GM	Superior temporal gyrus (PL)
Cingulate gyrus, anterior part right GM	Cingulate gyrus (AR)
Cingulate gyrus, anterior part left GM	Cingulate gyrus (AL)
Cingulate gyrus, posterior part right GM	Cingulate gyrus (PR)
Cingulate gyrus, posterior part left GM	Cingulate gyrus (PL)
Frontal lobe right GM	Frontal cortex (R)
Frontal lobe left GM	Frontal cortex (L)
Parietal lobe right GM	Parietal cortex (R)
Parietal lobe left GM	Parietal cortex (L)
Caudate nucleus right	Caudate nucleus (R)
Caudate nucleus left	Caudate nucleus (L)
Thalamus right, high intensity part in T2	Thalamus (R)
Thalamus right, low intensity part in T2	Thalamus (R)
Thalamus left, high intensity part in T2	Thalamus (L)
Thalamus left, low intensity part in T2	Thalamus (L)

**Frontal lobe**	**Parietal lobe**
Precentral gyrus	Postcentral gyrus
Superior frontal gyrus D	Superior parietal lobule
Middle frontal gyrus	Inferior parietal lobule
Inferior frontal gyrus O	Supramarginal gyrus
Inferior frontal gyrus T	Angular gyrus
SMA	
Superior frontal gyrus M	

Table S2: Frontal and parietal brain regions extracted from the UNC atlas (Shi et al., 2011). D, O, T and M for dorsal, opercular, triangular and medial respectively. SMA refers to Supplementary Motor Area.

PMW	N	Silhouette Score	Cov_IoU - subjects	Cov_IoU - time	Verdict
37	32	4>~5>~6>~7	3 ~ 4 and 6 >~7		4
38	46	4>~5>~6>~7 & std5 low	3~4~5 and 6>5		4 or 5
				Big decrease 38->39	
39	70	3>~4>~5>~6>~7 & std3,5,7 low	4 ~5 and 6~7 and 6>>5	5=6	4 or 5
40	104	3>~4>~5>~6>~7 (6=~7)	3>4	7>~6	5
41	153	4>>5 and 5>6>7 and std5 vlow	4=5 and 7>>6	5>>4>~3	4 (or 5)
				Decrease 41->42	
42	135	3>>4 and 4>~5>~6>~7 & std5 low	4>5 and 6> 5		5 (or 6)
43	103	3=4=5 and ~5>~6>~7 & std4 low	3>4		5
44	87	4=5 and 5>>6 and 6>7	6~5 and 7>6	5=6	5 (or 6)

Figure S4: Heuristics on the choice of the number of clusters k for k-means in KNIT. The symbols "$=$", "$>$" and "$<$" signify in this context "similar", "better than" and "worse than", respectively. The last column contains the optimal k.

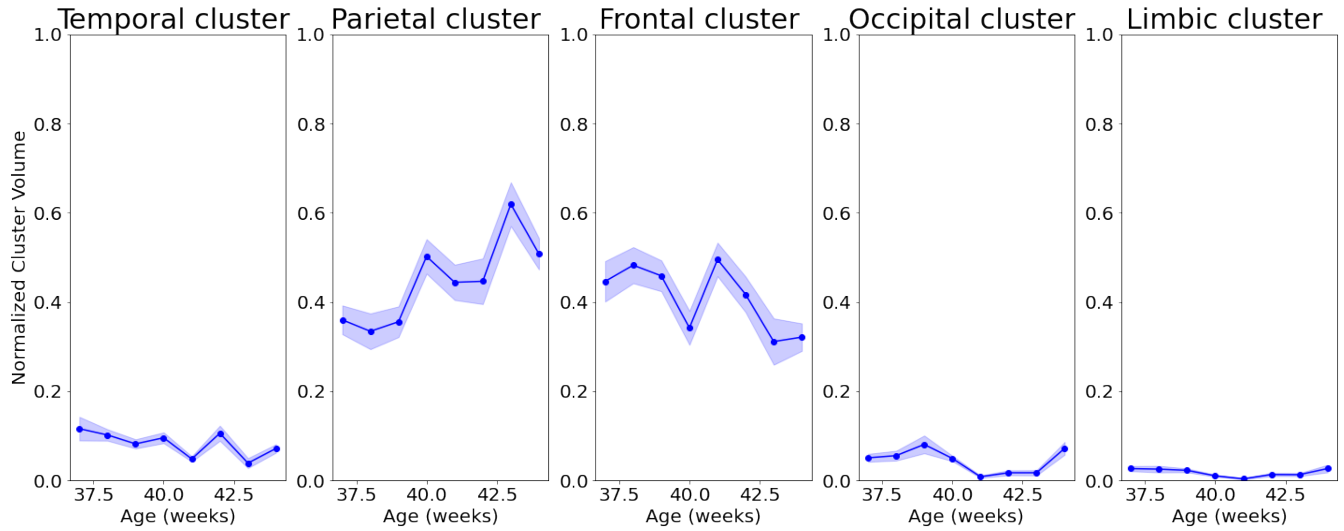

Figure S5: Developmental evolution of the normalized volumes (across whole thalamic volume) for each cluster, for the WTA approach. Mean and standard deviations across *subjects bootstraps* are displayed.

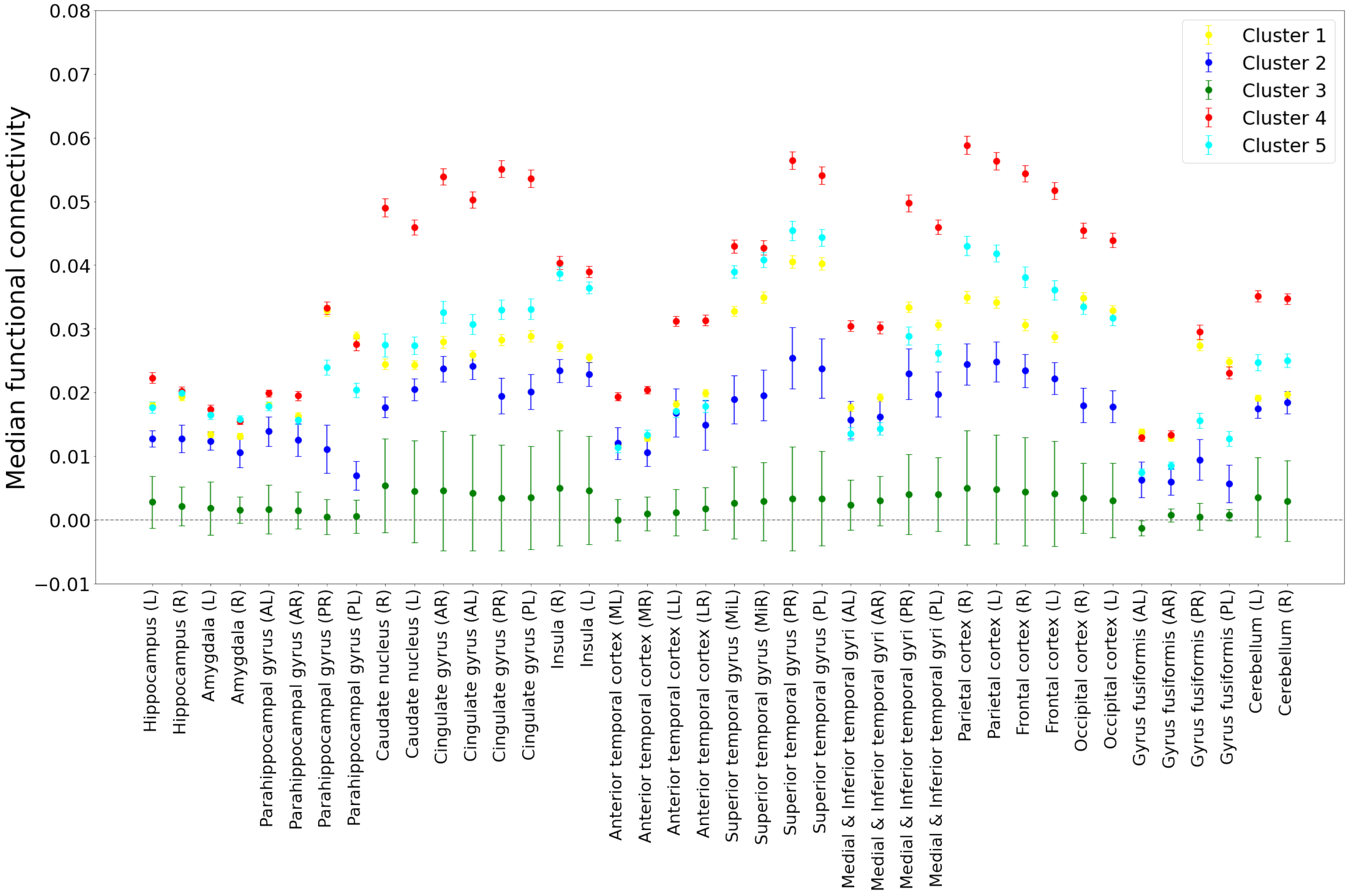

Figure S6: Median FC between each cluster ID and the 40 bilateral brain regions (ROIs). Mean and standard deviations are reported across *subjects bootstraps*, for the 44 weeks age group.

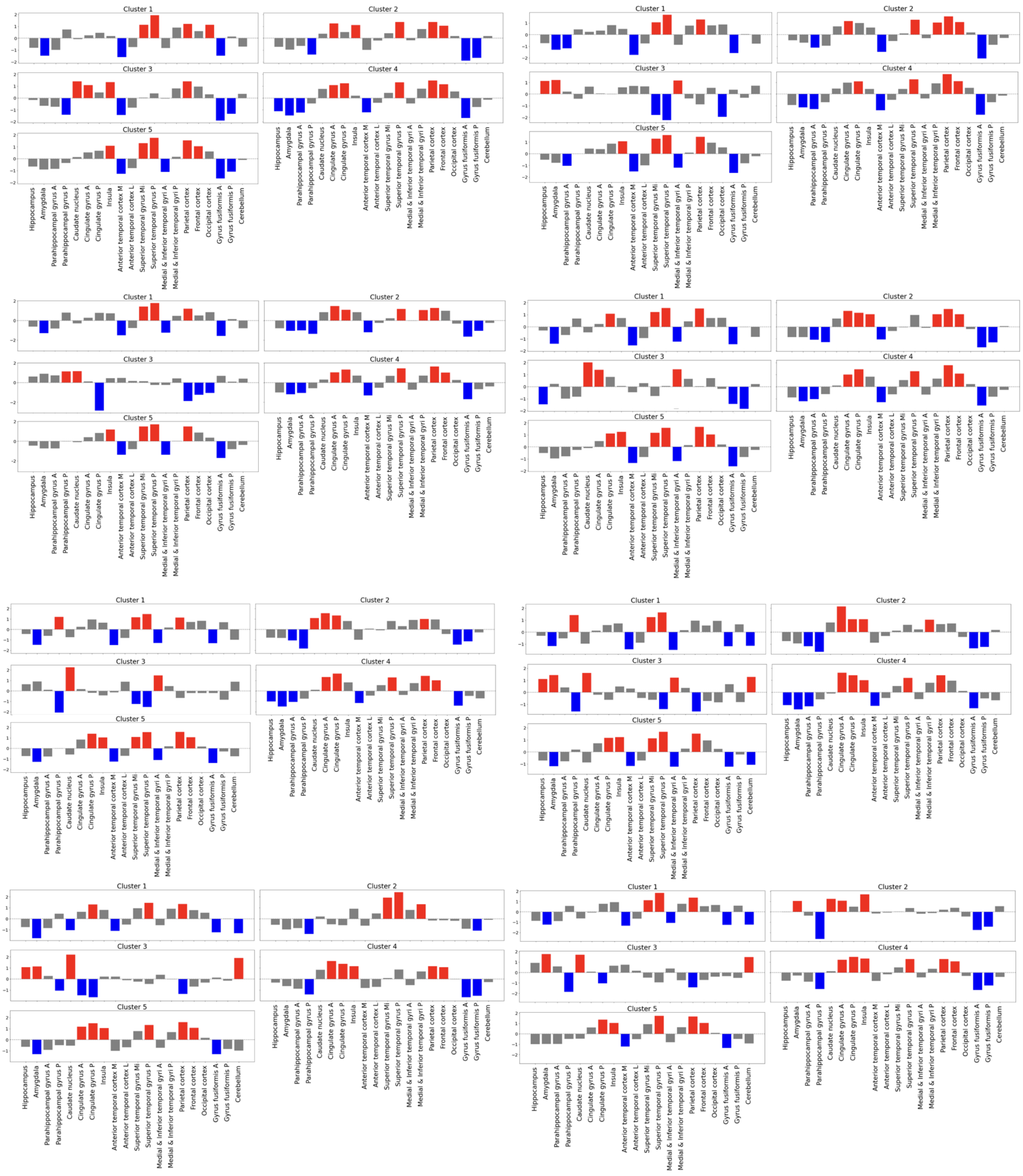

Figure S7: Z-score of the median FC, between each cluster ID and the 40 bilateral brain regions that were merged between the right and the left hemispheres, for all age groups. From 44 weeks on the top left to the 37 weeks age group on bottom right.

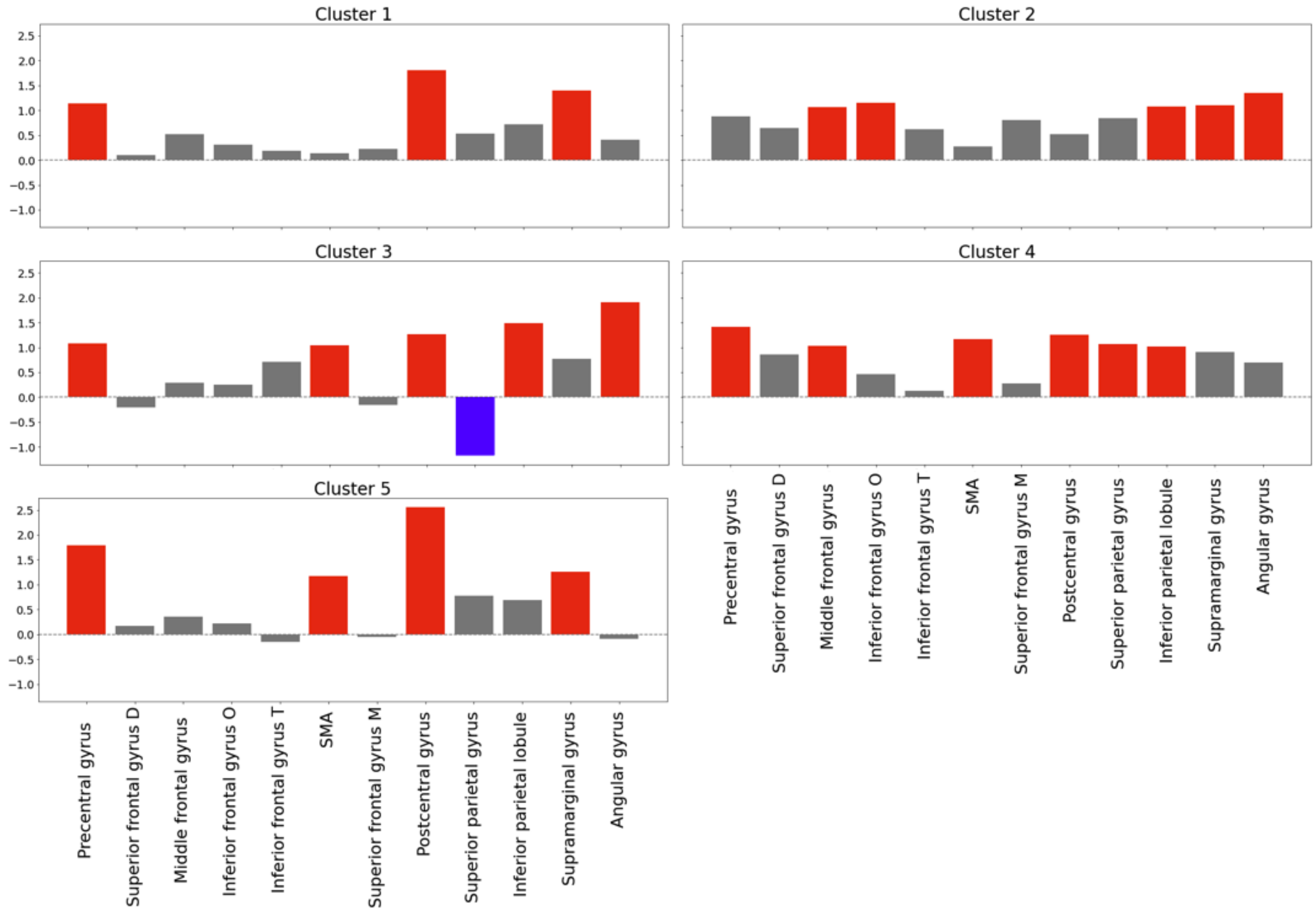

Figure S8: Z-score of the FC, between each cluster ID and the 12 bilateral brain regions (coming from frontal and parietal regions of UNC atlas (Shi et al., 2011)) that were merged between the right and the left hemispheres.

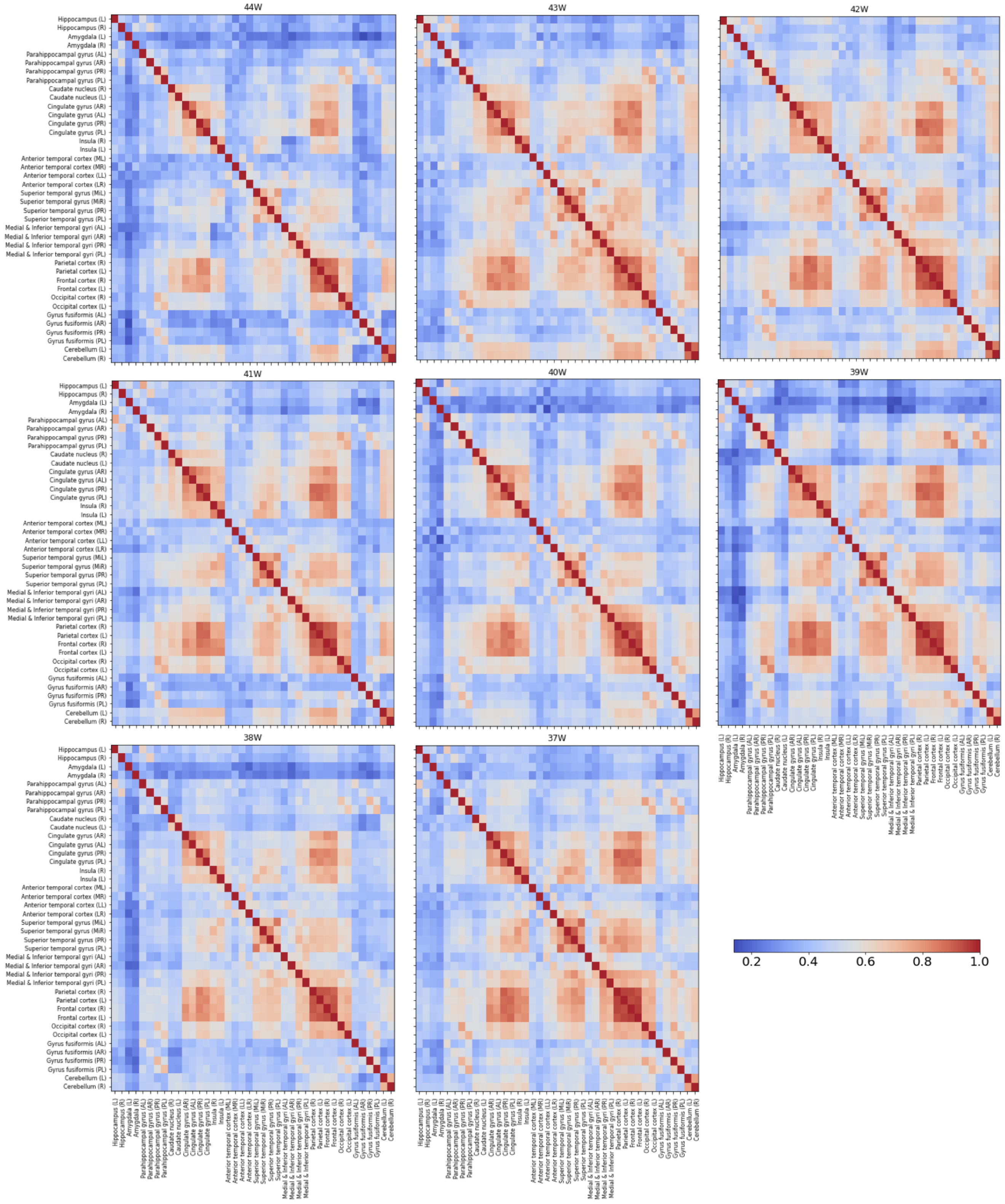

Figure S9: Functional connectivity matrices between the 40 ROIs of the different age groups.

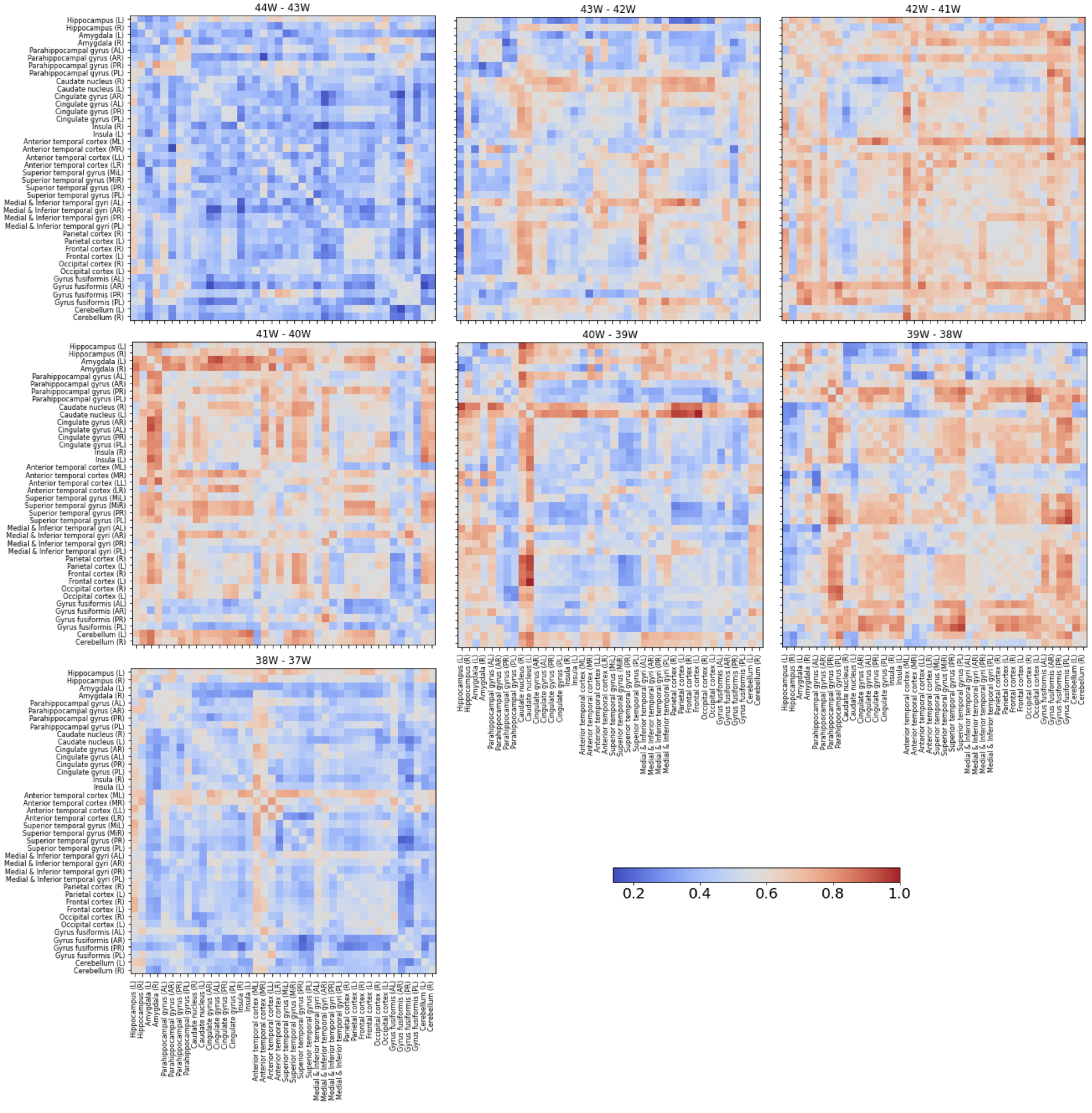

Figure S10: Functional connectivity matrix differences of consecutive age groups, between the 40 ROIs.

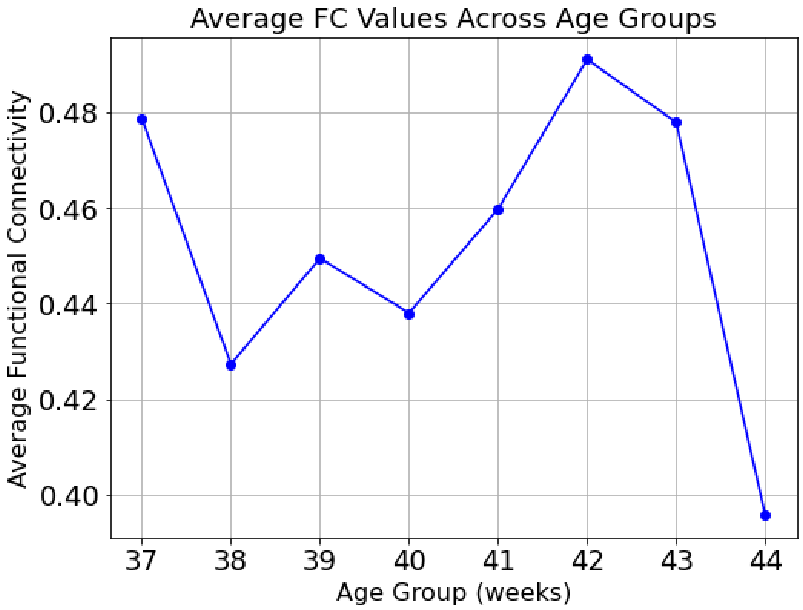

Figure S11: Average ROI-ROI functional connectivity evolution across different age groups.

References

Shi, F., Yap, P.-T., Wu, G., Jia, H., Gilmore, J. H., Lin, W., & Shen, D. (2011). Infant brain atlases from neonates to 1-and 2-year-olds. *PloS one*, *6*(4), e18746.
